# HBV Infection Drives PSMB5-Dependent Proteasomal Activation in Humanized Mice and HBV-Associated HCC

**DOI:** 10.3390/v17111454

**Published:** 2025-10-31

**Authors:** Ayse Tarbin Jannuzzi, Gulce Sari, Sema Arslan-Eseryel, Mujdat Zeybel, Yusuf Yilmaz, Murat Dayangac, Buket Yigit, Kazim Yalcin Arga, Andre Boonstra, Fatih Eren, Betul Karademir-Yilmaz

**Affiliations:** 1 Department of Pharmaceutical Toxicology, School of Pharmacy, Istanbul University, Istanbul 34116, Turkey; tarbin.cevik@istanbul.edu.tr; 2 Department of Biochemistry, School of Medicine & Genetic and Metabolic Disease Research and Investigation Center, Marmara University, Istanbul 34854, Turkey; g.sari@erasmusmc.nl (G.S.); semarsln1@hotmail.com (S.A.-E.); 3 Department of Gastroenterology and Hepatology, Erasmus Medical Center, 3015 GD Rotterdam, The Netherlands; p.a.boonstra@erasmusmc.nl; 4 Department of Gastroenterology and Hepatology, School of Medicine, Koc University, Istanbul 34450, Turkey; mzeybel@ku.edu.tr (M.Z.); byigit@ku.edu.tr (B.Y.); 5 Department of Gastroenterology, School of Medicine, Recep Tayyip Erdoğan University, Rize 53200, Turkey; dryusufyilmaz@gmail.com; 6 Department of General Surgery, International School of Medicine, Istanbul Medipol University, Istanbul 34810, Turkey; mdayangac@hotmail.com; 7 Department of Bioengineering, Marmara University, Istanbul 34854, Turkey; kazim.arga@marmara.edu.tr; 8 Department of Medical Biology, School of Medicine, Marmara University, Istanbul 34854, Turkey; fatiheren@marmara.edu.tr; 9 Department of Medical Biology, School of Medicine, Recep Tayyip Erdogan University, Rize 53200, Turkey; 10 Liver Research Unit, Institute of Gastroenterology, Marmara University, Istanbul 34854, Turkey; 11 Department of Biochemistry, School of Medicine, Recep Tayyip Erdogan University, Rize 53200, Turkey

**Keywords:** hepatitis B virus, hepatocellular carcinoma, ubiquitin-proteasome system, proteasome subunit β5

## Abstract

Hepatocellular carcinoma (HCC), the most common primary liver malignancy worldwide, is strongly associated with chronic Hepatitis B Virus (HBV) infection, a significant risk factor. The ubiquitin–proteasome system, central to protein degradation, cellular homeostasis, and cell cycle regulation, has been implicated in the pathogenesis of several cancers, including HCC. Despite this, the specific expression patterns of proteasomal subunits during HBV infection and HBV-induced HCC, as well as the association between mRNA expression of proteasomal subunits and proteasomal activity, remain poorly defined. To address this critical knowledge gap, we analyzed mRNA expression profiles of proteasomal subunits in HBV-infected humanized mouse models to uncover HBV-specific molecular alterations. Our findings revealed that the chymotrypsin-like activity (β5) subunit of the proteasome (PSMB5) is consistently overexpressed following HBV infection. Functional studies demonstrated that β5 deficiency decreases MHC I levels on the cell surface and leads to the accumulation of ubiquitinated proteins, establishing a direct link between β5 overexpression and increased proteasomal activity. Concordantly, HBV-infected patient livers—regardless of HCC status—displayed elevated β5 mRNA/protein levels and enhanced chymotrypsin-like activity. Additionally, analysis of Protein Atlas data revealed that elevated β5 mRNA expression correlates with poor clinical prognosis in HCC patients. In summary, this study highlights how HBV infection induces significant alterations in proteasome function by elevating β5 expression and activity in human and mouse livers. These findings underscore the critical role of proteasomal dysregulation in HBV-associated liver pathology and provide new insights into its involvement in HCC development. Understanding the interplay between HBV infection and proteasome dynamics offers a valuable avenue for the identification of novel therapeutic targets and biomarkers in HCC.

## 1. Introduction

The ubiquitin proteasomal system (UPS) is the main degradation pathway for redundant and non-naïve proteins. UPS consists of the central proteolytic complex 26S proteasome and complex enzymatic machinery that catalyze the attachment of ubiquitin chains to target proteins to be degraded [1]. The catalytic core of the 26S proteasome is called the 20S proteasome. The 20S proteasome is a cylindrical structure with four stacked rings forming a central channel for protein degradation. It consists of 28 protein subunits: the two outer rings contain seven α (PSMA1–7) subunits each, while the two inner rings contain seven β (PSMB1–7) subunits each. Three different β subunits can hydrolyze peptide substrates with chymotrypsin-like β5 (PSMB5), trypsin-like β2 (PSMB7) and caspase-like β1 activities (PSMB6) [2]. The 19S proteasome is referred to as the regulatory particle and it is responsible for the deubiquitination and unfolding of the target proteins and leading them to the catalytic part of the proteasome. The Regulatory particle base consists of PSMC1–6 and PSMD1, 2 and 4 and ADRM1 subunits. Regulatory particle lid subunits are PSMD3, PSMD6–8 and PSMD11–14. Proteolytic degradation by the 26S proteasome is ubiquitination- and ATP- dependent while 20S proteasome is able to degrade proteins without the need of ubiquitination and ATP. Mainly oxidized proteins are rapidly degraded by the 20S proteasome [3]. Through protein degradation, the UPS plays a crucial role in regulating essential cellular pathways, including cell signaling, cell cycle regulation, cell differentiation, immune response, and neuronal activity [4,5,6]. Today several diseases such as cancers, neurodegenerative diseases and immune system-related disorders are known to be caused by malfunction of UPS components [7].

HBV is a small, enveloped DNA virus that attacks the liver and establishes a lifelong chronic infection. The tolerogenic properties of the liver and the non-cytopathic nature of HBV make it difficult to completely clear the virus by the host immune system [8,9]. The WHO estimates 1.2 million new infections each year and 254 million people were estimated to be living with chronic HBV (CHB) infection in 2022. In 2022, 1.1 million deaths were ascribed to HBV and HBV-related liver complications such as hepatocellular carcinoma (HCC) [10]. HCC is the most common liver malignancy, and the chance of developing HCC is 10–25-fold higher in chronically HBV infected patients [10,11,12]. HBV replication and release heavily depend on host factors, and HBV regulates cellular pathways selectively. One of these altered pathways is the proteasomal system. It has been demonstrated by several research groups that HBV proteins are selectively degraded by UPS and HBV is capable of escaping innate immune recognition via altering UPS targets [13,14,15]. However, a systematic approach to study HBV- and HBV-induced HCC-dependent changes in the expression and activity of proteasomal subunits are still missing [16,17]. Thus, the current study aims to fill this knowledge gap using highly relevant models like HBV-infected, liver-humanized mouse models [18,19] and also HBV-infected HCC and non-HCC patient liver and blood samples.

Herein, we report that HBV infection results in an increase in PSMB5 subunit levels in the human primary hepatocytes of a liver-humanized mouse model. PSMB5 gene-deficient THP-1 cells showed that PSMB5 levels affect the cell surface MHC I levels, and also intracellular ubiquitinated protein levels. Our major findings are that both liver tissue and serum samples of HBV infected and HBV-induced HCC patients have higher levels of PSMB5 which is supported by lower ubiquitinated and carbonylated protein levels. Our results suggest that HBV-induced upregulation of PSMB5, which persists during HCC development, may alter proteostasis and MHC I presentation, thereby shaping immune recognition in hepatocytes and potentially influencing disease progression.

## 2. Materials and Methods

### 2.1. Patients and Sample Collection

Liver tissue and serum samples were obtained from 10 patients with non-viral HCC, 10 patients with chronic HBV infection, and 10 patients with HBV-induced HCC, who underwent liver biopsy or transplantation surgery at Koç University Hospital and Istanbul Medipol Hospital between 2015 and 2021. HCC diagnosis was confirmed by histopathological examination. Liver tissues were immediately snap-frozen in liquid nitrogen and stored at −80 °C together with plasma samples until further analysis. Patients who had received chemotherapy or radiotherapy prior to surgery were excluded. In addition, four patients with HCV or HDV co-infection and two patients with insufficient tumor tissue (<30% tumor content in resected material by immunohistochemistry) were excluded. As controls, serum samples from 10 healthy individuals without known liver disease were included. Detailed demographic characteristics of each group are presented in Table 1. The study was approved by the Ethics Committees of Marmara University (Protocol No. 09.2018.552) and Koç University (2015.053.IRB1.014, 2017.139.IRB2.048, 2016.024.IRB2.005), and written informed consent was obtained from all participants.

### 2.2. Preparation of Human Liver Tissue Lysates

Human liver tissue lysates were prepared in lysis buffer (250 mM sucrose, 25 mM HEPES, 10 mM MgCl_2_, 1 mM EDTA, and 1.6 mM DTT). Tissues were homogenized with homogenization beads using a Bullet Blender tissue homogenizer (Next Advance, Troy, NY 12180, USA). The homogenates were centrifuged at 13,400× *g* for 30 min, and the resulting supernatants were collected for downstream assays. Protein concentrations were determined with the Bio-Rad protein assay kit (Bio-Rad Laboratories, Hercules, CA, USA) according to the manufacturer’s instructions.

### 2.3. Assessment of Proteasome Activity in Patient Liver and Serum Samples

The chymotrypsin-like activity (β5 subunit) of the proteasome was measured using the fluorogenic substrate Suc-Leu-Leu-Val-Tyr-7-amido-4-methylcoumarin (Suc-LLVY-AMC) (Sigma Aldrich, St. Louis, MO, USA). Aliquots (10 µL) of patient liver lysates or serum samples from patients and healthy controls were incubated in a reaction mixture containing 225 mM Tris, 45 mM KCl, 7.5 mM MgCl_2_, 7.5 mM magnesium acetate, and 1 mM DTT (final volume: 110 µL) for 10 min at room temperature. Subsequently, 10 µL of 20 mM Suc-LLVY-AMC was added, and samples were incubated at 37 °C for 30 min. Fluorescence of released AMC was measured using EnSpire Multimode Plate Reader (PerkinElmer, Waltham, MA, USA) at 360 nm excitation and 460 nm emission. Proteasome activity was calculated from a free AMC standard curve and normalized to protein concentration and incubation time.

### 2.4. Ubiquitin, Protein Carbonyl, and Proteasome β1/β2/β5 Subunit Levels in Liver and Serum Samples

Levels of ubiquitin, protein carbonyls, and proteasome β1, β2, and β5 subunits in patient liver lysates and in serum samples from patients and healthy controls were measured using commercial ELISA kits. Specifically, the PSMB5 ELISA kit (Cat. No. E-EL-H1902, Elabscience, Houston, TX, USA) and the Ubiquitin, Protein Carbonyl, and Proteasome β1/β2 kits (Bioassay Technology Laboratory, Shanghai, China) were used. Assays were performed according to the manufacturers’ instructions. Results for liver tissue lysates were normalized to protein concentration. Detailed information on all reagents, antibodies, and assay kits, including catalog numbers and suppliers, is provided in Supplementary Table S1.

### 2.5. Mice, Transplantation, and HBV Infection

Urokinase-type plasminogen activator (uPA)/NOD/Shi-scid/IL2Rγnull (NOG) mice were obtained from the Central Institute for Experimental Animals (Kawasaki, Japan) and bred at the Erasmus Medical Center Animal Facility. Zygosity was confirmed as previously described [20,21]. Mice were housed in ventilated cages (maximum four per cage) with free access to chow and water. Male mice aged 4–6 weeks (*n* = 8) were anesthetized and received intra-splenic injections of 0.5–2 × 10^6^ commercially available cryopreserved human hepatocytes (Lonza, Basel, Switzerland; Corning, Corning, NY, USA). Graft engraftment was confirmed by measuring human albumin levels in mouse serum using ELISA [22,23]. The study protocol was approved by the Erasmus Medical Center Animal Ethics Committee (DEC No. 141–12-11).

At eight weeks post-transplantation, successfully engrafted human-liver chimeric mice (*n* = 4) were intravenously inoculated with 200 µL of patient serum containing HBV genotype A (7.7 log IU/mL). Following inoculation, mice were housed individually. HBV viral load in serum and liver was determined using a dual-target qPCR approach with primers and probes specific to the preS and X genes [24,25].

### 2.6. Immunohistochemical Analysis of Mouse Liver

Transplanted mouse livers were fixed in 4% formaldehyde (Merck-Millipore, Darmstadt, Germany) and processed for standard hematoxylin and eosin (H&E) staining. To visualize transplanted human hepatocytes, sections were incubated with a mouse anti-human mitochondria antibody (Merck, Darmstadt, Germany). Antigen–antibody complexes were detected using 3,3′-diaminobenzidine (DAB; Dako, Copenhagen, Denmark) as chromogen, and slides were counterstained with hematoxylin.

### 2.7. Cell Culture and CRISPR-Cas9 Knockout

THP-1 cells (ATCC, TIB-202) were cultured in RPMI-1640 medium (Gibco, Thermo Fisher Scientific, Waltham, MA, USA) supplemented with 10% Fetal Bovine Serum (FBS; HyClone, Cytiva, Marlborough, MA, USA), 1% non-essential amino acids (NEAA; Gibco), 1% HEPES (Gibco), and 1% Antibiotic-Antimycotic (Gibco) at 37 °C in 10% CO_2_. For CRISPR-Cas9–mediated knockout, cells were transduced with lentiviral vectors and selected in Blasticidin (10 µg/mL; InvivoGen, San Diego, CA, USA) for 7 days. Knockout efficiency was assessed using the ICE CRISPR Analysis Tool (Synthego).

The plasmid targeting the human PSMB5 gene (#117073) and the LentiCRISPRv2 plasmid with blasticidin selection (#83480) were obtained from Addgene (Watertown, MA, USA). An unmodified plasmid was used as the empty vector (EV) control. Chemically competent bacteria were transformed by heat shock and cultured overnight at 37 °C on LB agar plates containing 100 µg/mL ampicillin. Plasmids were isolated from individual colonies using the Clontech plasmid isolation kit and sequenced by Genewiz-Azenta to confirm sgRNA insertion, with the primer hU6-F (5′-GAGGGCCTATTTCCCATGATT-3′). Following CRISPR-Cas9 targeting, sgRNAs and genomic DNA sequences from transduced cells were analyzed for insertion/deletion (indel) efficiencies using the ICE CRISPR Analysis Tool (Synthego, Menlo Park, CA, USA).

### 2.8. RNA Extraction and Real-Time qPCR

Mouse liver tissue was preserved in RNAlater Stabilization Solution (Qiagen, Hilden, Germany; Cat. No. 76106), whereas THP-1 cells were processed immediately after collection. Total RNA was extracted using the RNeasy Mini Kit (Qiagen, Hilden, Germany; Cat. No. 74104) and treated with DNase I RNase-Free DNase Set (Qiagen, Hilden, Germany; Cat. No. 79254) to remove genomic DNA contamination. cDNA was synthesized using the RNA to cDNA EcoDry Premix (Takara Bio, Kusatsu, Shiga, Japan; Cat. No. 639549) according to the manufacturer’s instructions. Human-specific gene expression was assessed by quantitative PCR with SYBR Green detection. Primer sets (Integrated DNA Technologies, Coralville, IA, USA) are listed in Table 2. Gene expression levels were normalized to GAPDH using the 2^−ΔCt method, where ΔCt = Ct_target − Ct_GAPDH. cDNA derived from non-chimeric mouse livers was used as a negative control to assess cross-reactivity of housekeeping and target gene primers.

### 2.9. Western Blot Analysis

THP-1 cell lysates were prepared in RIPA buffer supplemented with protease inhibitors (Pierce, Thermo Fisher Scientific, Waltham, MA, USA; Cat. No. A32955), and protein concentrations were determined using BCA Protein Assay Kit (Pierce, Thermo Fisher Scientific, Waltham, MA, USA; Cat. No. 23225). Denatured protein samples (30 μg) were separated on 10% SDS–PAGE gels (GenScript, Piscataway, NJ, USA; Cat. No. M00655) under reducing conditions and transferred to PVDF membranes (Millipore, Burlington, MA, USA; Cat. No. IPVH00010). Membranes were blocked in TBS-T (1×) containing 5% milk and incubated overnight at 4 °C with rabbit anti-human PSMB5 (BML-PW8895-0025, Enzo Life Sciences, Farmingdale, NY, USA) or rabbit anti-ubiquitin (07-375, Sigma Aldrich, St. Louis, MO, USA), diluted in TBS-T (1×) with 2% milk. The following day, membranes were washed three times in TBS-T (1×) and incubated for 1 h at room temperature with HRP-conjugated goat anti-rabbit secondary antibody (MilliporeSigma, Burlington, MA, USA; Cat. No. AP132P). After three further washes, signals were developed using HRP substrate (MilliporeSigma, Burlington, MA, USA; Cat. No. WBLUF0100). Membranes were subsequently stripped, re-blocked, and re-probed overnight at 4 °C with mouse anti-β-actin (Santa Cruz Biotechnology, Dallas, TX, USA, cat no. sc-47778) in TBS-T (1×) with 2% milk. Detection was carried out as described above.

### 2.10. Flow Cytometry of THP-1 Cell Surface Markers

THP-1 cells were stained with Zombie Violet Fixable Viability Kit (BioLegend, San Diego, CA, USA) to exclude dead cells. For surface MHC class I detection, cells were incubated with W6/32 hybridoma supernatant, followed by secondary staining with donkey anti-mouse Alexa Fluor 647 (Life Technologies, Carlsbad, CA, USA). Geometric mean fluorescence intensity (MFI) values were calculated for quantification, and data were analyzed and visualized using FlowJo™ software (v10.8.1; FlowJo LLC, Ashland, OR, USA).

### 2.11. Comparative Analysis of Gene Expression

Gene expression profiles of HCC samples were retrieved from The Cancer Genome Atlas (TCGA, https://www.cancer.gov/tcga, accessed on 13 September 2023). Samples were stratified by disease status (HCC vs. non-HCC) and HBV infection status (positive vs. negative). The final dataset comprised 15 HBV-positive HCC samples, 226 HBV-negative HCC samples, and 28 HBV-negative non-HCC (healthy) liver samples. Comparative analyses were performed using FPKM-normalized gene expression values.

### 2.12. Statistical Analyses

Results are expressed as mean ± standard deviation (SD). Statistical analyses were performed using GraphPad Prism version 7 (GraphPad Software, San Diego, CA, USA). Differences between groups were evaluated with the Mann–Whitney test, and a *p*-value < 0.05 was considered statistically significant.

## 3. Results

### 3.1. Higher Proteasomal Subunit Expression in HCC Is Associated with Poorer Survival

**Figure 1:**
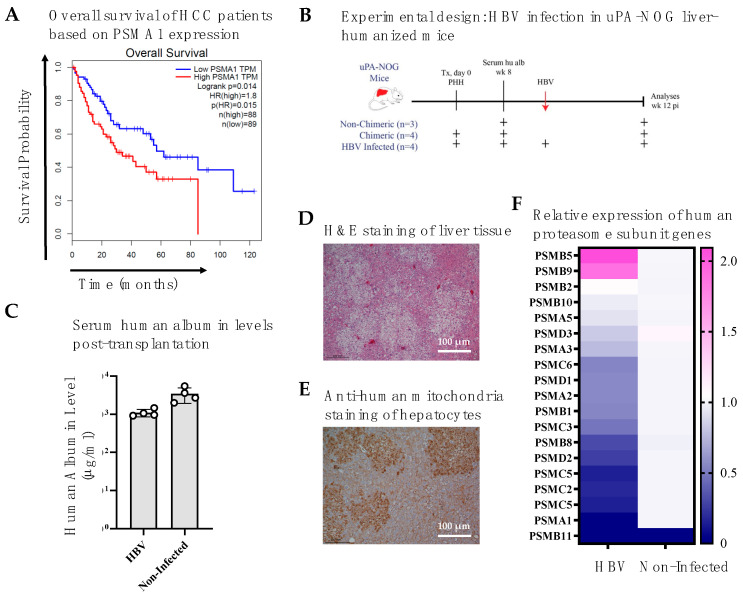
HBV infection enhances PSMB5 expression in liver-humanized mice. (**A**) 10-year survival analysis of low (bottom 75% of TPM) and high (top 25% of TPM) PSMA1 expressing HCC patients using GEPIA: a web server for cancer and normal gene expression profiling and interactive analyses [27]. HBV infection increases PSMB5 gene expression levels of liver humanized mice livers. (**B**) Schematic overview of HBV infection in uPA-NOG liver-humanized mice. (**C**) Human albumin levels in mouse serum, 8 weeks post-transplantation (**D**) H&E staining of mouse liver tissue. (**E**) Anti-human mitochondria staining indicating engrafted human hepatocytes after 12 weeks of infection. (**F**) Relative mRNA expression of 26S proteasome subunits in livers of HBV-infected vs. non-infected mice (*n* = 4/group). TPM: Transcripts per million. Scale bar: 100 μm.

The prognostic roles of proteasomes in cancer have been reported to vary across cancer types. To assess whether differential expression of proteasomal subunits impacts survival in HCC, we analyzed mRNA expression data from The Human Protein Atlas [26]. As noted earlier, the 20S core of the proteasome consists of PSMA1–7 and PSMB1–7 subunits, while the 19S regulatory particle includes PSMC1–6 and PSMD1–3. Strikingly, expression of nearly all 26S proteasome subunits was significantly negatively correlated with 5-year overall survival in HCC patients (Table 3). In other words, patients with lower proteasomal subunit mRNA expression exhibited better 5-year survival. As a representative example, Figure 1A illustrates PSMA1 transcript-per-million (TPM) expression in relation to the 10-year survival probability of HCC patients.

### 3.2. HBV Infection in Liver-Humanized Mice Increases PSMB5 Transcript Levels

To investigate the effect of HBV infection on proteasomal subunit expression dynamics, we used a human-liver chimeric mouse model (Figure 1B). Non-transplanted uPA-NOG mice (*n* = 3) served as negative controls, whereas transplanted mice were divided into HBV-infected (*n* = 4) and non-infected (*n* = 4) groups. Successful engraftment of primary human hepatocytes into uPA-NOG mouse livers was confirmed by high serum human albumin levels at week 8 post-transplantation (Figure 1C). Engrafted mice were then infected with patient serum containing HBV genotype A, and livers were collected 12 weeks post-infection for further analysis.

**Figure 2:**
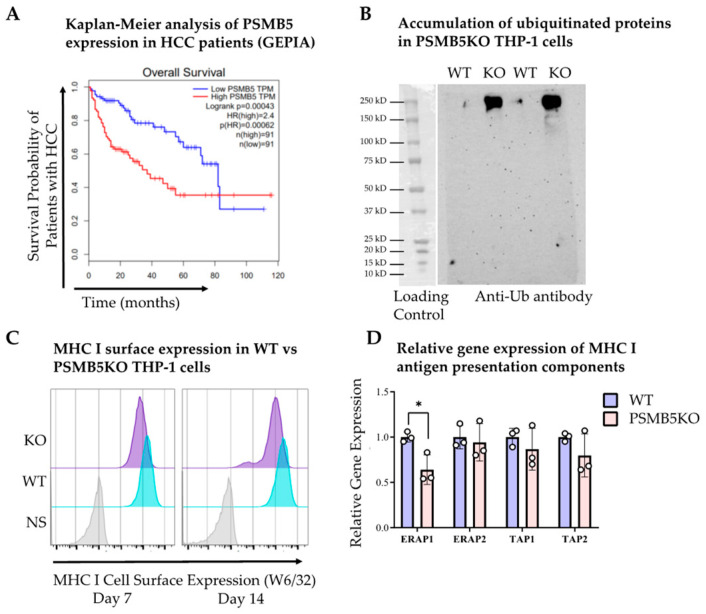
Functional consequences of PSMB5 knockout in human THP-1 cells. (**A**) Ten-year survival analysis of low (bottom 75% of TPM) and high (top 25% of TPM) PSMB5 expressing HCC patients using GEPIA: a web server for cancer and normal gene expression profiling and interactive analyses [27]. (**B**) PSMB5KO cells accumulate ubiquitinated proteins due to decreased protein degradation. Western blot analysis showing accumulation of ubiquitinated proteins in PSMB5-deficient (KO) THP-1 cells compared with wild-type (WT) controls on day 7. (**C**) Flow cytometric analysis of MHC I surface expression in WT and PSMB5KO THP-1cells at days 7 and day 14 post-transduction. (**D**) Relative mRNA expression levels of antigen-processing pathway genes in WT (*n* = 3) and PSMB5-KO (*n* = 3) THP-1 cells. WT: wild-type control; KO: PSMB5 knockout. * *p* < 0.05.

Histological assessment with H&E staining (Figure 1D) and immunohistochemistry using anti-human mitochondria antibody (Figure 1E) confirmed the presence of human hepatocyte islands in the livers at 12 weeks (scale bar = 100 μm). qPCR analysis of human 26S proteasome subunit transcripts revealed a >2-fold increase in PSMB5 expression in HBV-infected mice compared to non-infected controls (Figure 1F). Consistent with PSMA1, higher PSMB5 expression correlated with poorer overall survival in HCC patients, particularly within the first 5 years (Figure 2A).

These findings indicate that HBV infection directly activates the proteasomal machinery in human hepatocytes, potentially reflecting a viral strategy to enhance protein turnover and escape immune detection. The marked upregulation of PSMB5 in infected livers suggests that proteasome hyperactivity is an early molecular event in HBV-driven hepatocarcinogenesis.

Since PSMB5 encodes the β5 (chymotrypsin-like) catalytic subunit, which plays a crucial role in antigen processing and is a known therapeutic target of proteasome inhibitors [28], we next generated PSMB5-deficient THP-1 cells to study its function (Figure 2B). PSMB5 knockout (KO) cells accumulated ubiquitinated proteins and showed a modest reduction in MHC I surface expression compared with wild-type (WT) control cells (KO GMFI: 7.06 × 10^3^ vs. WT GMFI: 1.96 × 10^4^, Figure 2C). This reduction is likely due to a depleted antigen pool [29], as expression of most antigen-processing components was comparable to wild-type cells, with the exception of ERAP1 (Figure 2D). Similarly, TCGA data indicated that antigen presentation pathway gene expression did not differ significantly between HBV-infected and HBV-negative HCC patient livers (Supplementary Figure S1).

The accumulation of ubiquitinated proteins and modest decrease in MHC I surface levels in PSMB5-deficient cells further support a mechanistic link between β5 activity and efficient antigen processing. This finding underscores the importance of PSMB5 in maintaining cellular proteostasis and immune surveillance. Enhanced proteasome function during HBV infection may thus facilitate viral persistence and immune evasion, contributing to chronic infection and oncogenic progression.

In summary, HBV infection upregulates PSMB5 expression in liver-humanized mice, and high PSMB5 levels are associated with poorer survival in HCC. Given its role in protein turnover and antigen presentation, PSMB5 may contribute mechanistically to HBV-driven hepatocarcinogenesis.

### 3.3. HBV-Infected Patient Livers Exhibit Elevated Chymotrypsin-like Proteolytic Activity

**Figure 3:**
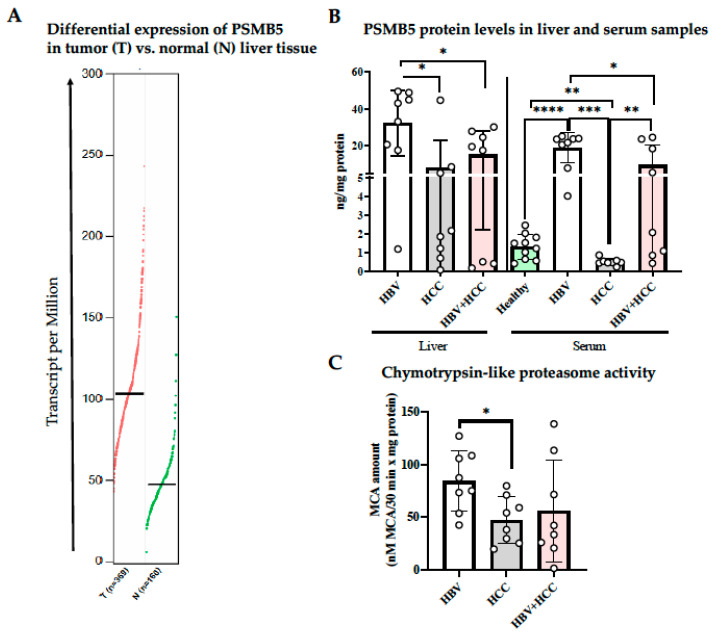
Elevated β5 subunit expression and proteasome activity in HBV-infected human tissues. (**A**) Differential expression of PSMB5 gene in tumor and corresponding normal tissue from patients with HCC using GEPIA [27]. HBV infected patients have higher levels of β5 protein expression and proteasome activity levels. Liver and serum samples of HBV, HCC and HBV-induced HCC patients were collected; (**B**) proteasome subunit β5 protein expression and (**C**) proteasome activity were analyzed with ELISA methods. * *p* < 0.05, ** *p* < 0.01, *** *p* < 0.001, **** *p* < 0.0001.

In addition to its association with poor survival in HCC, PSMB5 mRNA expression was significantly higher in tumor tissues of HCC patients compared with matched non-tumor liver tissues [27] (Figure 3A). This prompted us to investigate whether elevated transcript levels translate into increased proteasome activity and whether such changes are influenced by HBV infection status.

Protein levels of the proteasome β5 subunit were measured in fresh liver tissues and serum samples from patients with chronic HBV infection, non-viral HCC, and HBV-positive HCC using ELISA. Serum samples from healthy individuals were included for comparison. Both HBV-infected HCC and HBV-infected non-HCC liver samples showed increased PSMB5 protein expression, which was also reflected in patient serum samples (Figure 3B). Importantly, elevated PSMB5 protein expression corresponded with increased chymotrypsin-like proteolytic activity: chronic HBV-infected non-HCC patient livers displayed significantly higher proteasome activity compared with non-viral HCC livers (Figure 3C).

**Figure 4:**
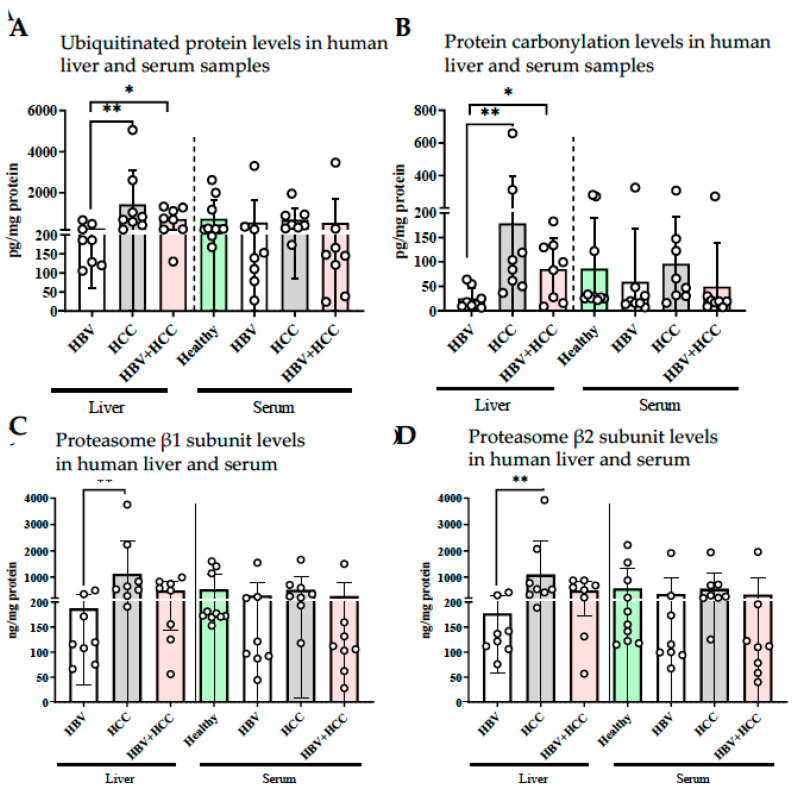
HBV infected, non-HCC patients have lower levels of ubiquitinated and carbonylated protein accumulation. Liver and serum samples of HBV, HCC and HBV-induced HCC patients were collected and (**A**) ubiquitin levels; (**B**) protein carbonylation levels; (**C**) proteasome subunit β1 protein expression levels and (**D**) proteasome subunit β2 protein expression levels were analyzed with ELISA methods. * *p* < 0.05, ** *p* < 0.01.

This transcriptional upregulation was accompanied by reduced levels of tissue ubiquitin and protein carbonylation in HBV-infected non-HCC livers (Figure 4A,B). Although HBV-infected HCC livers also showed lower protein carbonyl levels, the differences were not statistically significant. In addition, HBV-infected non-HCC livers exhibited significantly reduced protein levels of the β1 and β2 proteasome subunits, whereas no significant differences were observed between viral and non-viral HCC groups (Figure 4C,D).

Collectively, these results indicate that HBV infection promotes a hyperactive proteasome phenotype in hepatocytes, reducing oxidative stress and misfolded protein burden. While such adaptation may initially protect hepatocytes from proteotoxic damage, persistent proteasome activation may paradoxically create a pro-tumorigenic environment by supporting metabolic flexibility, immune escape, and survival under stress. These features are consistent with the observed association between high PSMB5 expression and poor patient prognosis in HBV-related HCC.

## 4. Discussion

Hepatitis B virus (HBV) infection is a major driver of hepatocellular carcinoma (HCC) development, yet the molecular mechanisms connecting viral persistence to tumor progression remain incompletely understood. In this study, we identify the proteasomal subunit PSMB5 as a key mediator in this axis, demonstrating that HBV infection alters proteasome dynamics in hepatocytes and contributes to oncogenic transformation.

By focusing on the ubiquitin–proteasome system (UPS)—a central regulator of protein homeostasis, cell cycle progression, and immune surveillance—we show in patient liver samples, serum samples, and liver-humanized mouse models that HBV not only modulates the expression of proteasomal subunits but also enhances their enzymatic activity, particularly that of the chymotrypsin-like β5 subunit. This hyperactivation may generate a cellular environment that favors immune evasion, viral persistence, and ultimately hepatocarcinogenesis.

Our analyses demonstrate that mRNA expression levels of nearly all 26S proteasome subunits negatively correlate with 5-year survival in HCC patients, underscoring the prognostic implications of proteasomal upregulation. In particular, PSMA1 and PSMB5, representative subunits of the α and β rings of the 20S proteasome, showed strong associations with poorer survival. This suggests that proteasome hyperactivity may facilitate tumor adaptation in advanced disease stages by enhancing degradation of misfolded proteins and preventing stress-induced apoptosis. Given the established role of the proteasome in maintaining cellular homeostasis, it is not surprising that proteasome inhibition has emerged as a therapeutic strategy in oncology, with several inhibitors already approved for hematological malignancies and others under evaluation for solid tumors [30].

Using a human-liver chimeric mouse model, we observed that HBV infection significantly increases PSMB5 transcript and protein levels, accompanied by enhanced β5 proteolytic activity. This finding was reinforced in PSMB5-deficient THP-1 cells, which exhibited reduced MHC I surface expression and accumulation of ubiquitinated proteins. Together, these data highlight how HBV-induced PSMB5 upregulation may impair antigen presentation, promote viral persistence, and facilitate oncogenic transformation.

Interestingly, serum and tissue analyses revealed higher PSMB5 levels in HBV-infected individuals irrespective of HCC status, whereas non-viral HCC patients had comparatively lower levels. Notably, PSMB5 elevation was more pronounced in HBV-infected non-tumor livers than in HCC tissues, suggesting that viral infection itself is a stronger driver of proteasome activation than malignant transformation. This raises the possibility that HBV-induced proteasomal changes represent an early tumorigenic event, priming hepatocytes for transformation by modulating protein degradation and immune signaling pathways. Circulating PSMB5 may thus hold potential as a liquid biopsy marker for detecting early HBV-induced liver alterations, a hypothesis that warrants validation in larger cohorts.

In addition, HBV infection was associated with reduced levels of ubiquitinated and carbonylated proteins in non-HCC livers, consistent with enhanced protein turnover. While this adaptation may initially mitigate oxidative stress and protein misfolding, sustained proteasome hyperactivity may ultimately support malignant transformation by enabling rapid growth and survival of hepatocytes.

Earlier studies investigating HBV–UPS interactions have largely focused on the HBx protein, which plays an important role in HBV-related hepatocarcinogenesis [31]. HBx itself is degraded by the proteasome through both ubiquitin-dependent and -independent pathways [32], and several viral proteins are targeted by UPS degradation, linking proteasome activity to HBV replication [32,33,34,35,36]. Our results differ from earlier reports, such as Hu et al. (1999), who observed reduced β5 activity in HBx-expressing HepG2 cells [35]. This discrepancy may reflect limitations of immortalized cell lines, which lack essential components of the immune microenvironment, whereas our use of primary human hepatocytes, patient liver samples, and humanized mouse models provides a more physiologically relevant context.

The broader implications of our findings underscore the dual role of proteasome upregulation in HBV-infected livers: promoting viral persistence and providing tumor cells with a selective advantage under stress conditions. These insights suggest that proteasome-targeting therapies could hold promise in HBV-associated HCC. While current proteasome inhibitors such as bortezomib are approved for hematological malignancies, their efficacy in solid tumors remains under investigation. Selective β5 inhibitors, including carfilzomib and ixazomib, may offer improved specificity and tolerability, but their utility in HBV-associated HCC must be approached cautiously and requires rigorous clinical evaluation [37].

Further validation of the PSMB5-driven proteasome activation signature in independent HBV-related HCC cohorts from different geographic regions, including Asia and the Americas, would be highly valuable. Collaborative efforts across centers are currently underway to prospectively confirm these findings in larger, ethnically diverse patient populations.

Future studies should aim to clarify the causal relationship between HBV infection, proteasomal hyperactivity, and hepatocarcinogenesis. Proteomics-based approaches examining post-translational modifications of proteasome subunits in HBV-infected versus non-infected livers may uncover novel regulatory mechanisms. Additionally, exploring co-treatment strategies that combine proteasome inhibition with antiviral therapy could open new therapeutic avenues, potentially improving clinical outcomes in HBV-related HCC.

## 5. Conclusions

In conclusion, our study reveals that HBV infection upregulates the proteasomal subunit PSMB5 and enhances its β5 proteolytic activity, thereby altering protein turnover and antigen presentation. These findings provide mechanistic insight into HBV-driven hepatocarcinogenesis and suggest that proteasome dysregulation may be an early step in tumor progression.

## Figures and Tables

**Table 1:** Patient and healthy control demographics.

Characteristics	HBV (*n* = 8)	Non-HBV + HCC (*n* = 8)	HBV + HCC (*n* = 8)	Healthy Controls (*n* = 10)
Male/Female *n* (%)	6/2 (75.0)/(25.0)	6/2 (75.0)/(25.0)	8/0 (100.0/0.0)	5/5 (50.00/50.00)
Age (±)	52.4 ± 7.5	53.8 ± 19.3	61.8 ± 6.0	47.6 ± 9.6
Cirrhosis *n* (%)	Yes 8 (100.0)	Yes 4 (75.00)	Yes 5 (62.5)	NA
ALT (±)	31.3 ± 30.3	39.1 ± 39.9	31.6 ± 7.3	20.8 ± 4.05
Ethnicity *n* (%)	White 8 (100.0)	White 8 (100.0)	White 8 (100.0)	White 10 (100.00)
Fibrosis Parameters				
FIB-4 Index (±)	6.8 ± 3.9	5.4 ± 2.2	3.9 ± 2.8	NA
ISHAK Score (%)	6 (100)	NA	NA	NA
Fibrosis Stage *n* (%) *				NA
F0–2	NA	1 (12.5)		
F3	NA	1 (12.5)	1 (12.5)	
F4	NA	4 (50.0)	5 (62.5)	

Data presented as mean ± SD. Categorical variables are given as frequencies (percentages). * In the HCC and HBV + HCC cohorts, two patients had missing data on their fibrosis stage.

**Table 2:** Primer combinations used for qPCR.

Target	Sequence	Target	Sequence
PSMA1-F	ATACTTTCGGCAGCACCTCC	PSMB10-R	CTGCGGTCCAGTCAGGTCTA
PSMA1-R	AGACCAACTGTGGCTGAACC	PSMB11-F	CGTGGCTATCGCTACGACAT
PSMA2-F	GTGCTTTGGCTCTTCGGGTA	PSMB11_R	TGACACATGCTCCCATCCAC
PSMA2-R	GCTTTAATTCCCACGGACGG	PSMC2-F	ACAGCCTTTACAGGTTGCCA
PSMA3-F	GGCTGCAGTTTCATGTTAGGG	PSMC2_R	CTATCCACGCCCACTCTCATC
PSMA3-R	GATGGCACAGCCCCAATAAC	PSMC3_F	ATTGGGGGTTTGGACAAGCA
PSMA5-F	GTACGACAGGGGCGTGAATA	PSMC3_R	ATCAGCACCCCTTTTGGAGG
PSMA5-R	GCACACACCCTCTGATGTCT	PSMC4_F	TCTGGAGGCTGTGGATCAGA
PSMB1-F	CCCTTTGCAGCTGCGATTTT	PSMC4_R	AGTGCATTGCTGTGCTTGTG
PSMB1-R	GGGCTATCCCGCGTATGAAT	PSMC5_F	AGAGAAGATGGCGCTTGACG
PSMB2-F	CTCATCGGTATCCAAGGCCC	PSMC5_R	CTCCGGAGGTTTTGGCTCTT
PSMB2-R	GTCTCCAGCCTCTCCAACAC	PSMC6_F	AACACAAGGAGATCGACGGC
PSMB5-F	GCTACCGGTGAACCAGCG	PSMC6_R	CGATCTGCCCAACACTCTGTA
PSMB5-R	CAACTATGACTCCATGGCGGA	PSMD1_F	ATGGGAGGATGGAAGAGGCT
PSMB8-F	GCTCCTGGCTGACTTCTAGT	PSMD1_R	AACATGTAGCAGGCGTCGAA
PSMB8-R	TGAACGTTCCTTTCTCCGTCC	PSMD2_F	CATGACTTCAGTGCCCAAGC
PSMB9-F	GGCGTTGTGATGGGTTCTGA	PSMD2_R	CGGAGATGATGTCAGCAGCA
PSMB9-R	AGAGAGTGCACAGTAGATGCG	PSMD3_F	GCGAATCAAAGCCATCCAGC
PSMB10-F	AGCTACACGCGTTATCTACGG	PSMD3_R	CAGCTCCACCACGATGAGAA

**Table 3:** The correlation between proteasomal subunit mRNA expression and patient survival in liver cancer using The Human Protein Atlas and Kaplan–Meier Analysis. Gene expression is analyzed as ‘Fragments Per Kilobase of Transcript per Million (FPKM)’ and based on the FPKM value of each gene, patients were automatically classified into two groups using the best expression cur-off values. The best expression cut-off refers to the FPKM value that yields maximal difference with regard to survival between the two groups at the lowest log-rank *p*-value.

Gene Name	5-Year Survival of High Expressers (%)	5-Year Survival of Low Expressers (%)	*p*-Value ^$^
PSMA1	36	56	0.000038
PSMA2	32	56	0.00013
PSMA3	37	57	0.002
PSMA5	42	57	0.00023
PSMA6	44	49	0.075
PSMA7	32	59	0.000025
PSMB1	39	51	0.024
PSMB2	31	56	0.00000035
PSMB3	43	59	0.055
PSMB4	39	55	0.00075
PSMB5	31	57	0.000029
PSMB6	43	52	0.27
PSMB7	44	50	0.02
PSMB8	34	55	0.078
PSMB9	40	56	0.057
PSMB10	50	43	0.072
PSMC1	42	53	0.012
PSMC2	41	51	0.021
PSMC5	43	52	0.0083
PSMC6	38	51	0.0031
PSMD1	19	58	8.6 × 10^−12^
PSMD2	32	53	0.00000066
PSMD3	43	51	0.0057

^$^ Low-rank *p* value for Kaplan–Meier plot showing results from analysis of correlation between mRNA expression level and patient survival.

## Data Availability

The gene expression profiles of HCC samples employed in the study are openly available in The Cancer Genome Atlas [TCGA, https://www.cancer.gov/tcga, accessed on 13 September 2023]. The original contributions presented in this study are included in the article/Supplementary Material. Further inquiries can be directed to the corresponding author(s).

## Supplementary Materials

The following supporting information can be downloaded at https://www.mdpi.com/article/10.3390/v17111454/s1. Figure S1: Differential expression of antigen processing and presentation genes in HBV-associated and non-HBV HCC. Table S1. Reagents, materials, and commercial sources. TCGA data indicated that antigen presentation pathway gene expression did not differ significantly between HBV-infected and HBV-negative HCC patient livers (Supplementary Figure S1). Gene expression levels were compared among HBV-positive HCC (*n* = 15), HBV-negative HCC (*n* = 226), and HBV-negative non-HCC (*n* = 28) groups using TCGA data. * *p* < 0.05, ** *p* < 0.01, *** *p* < 0.001.

## Abbreviations

The following abbreviations are used in this manuscript:

ALT	Alanine transaminase
APC	Antigen presentation complex
HBV	Hepatitis B virus
HCC	Hepatocellular carcinoma
CHB	Chronic hepatitis B
MHC	Major histocompatibility complex
NA	Not applicable
PSMB5	Proteasome 20S Subunit Beta 5
UPS	Ubiquitin proteasomal system
sgRNA	Single guide RNA
uPA-NOG	urokinase-type plasminogen activator- NOD/Shi-scid IL2Rγ^null^ mouse
