# Supplementary material for: HBV Infection Drives PSMB5-Dependent Proteasomal Activation in Humanized Mice and HBV-Associated HCC
Source: Viruses. 2025 Oct 31;17(11):1454. doi: 10.3390/v17111454 (PMC12656990; doi:10.3390/v17111454)

Expression of antigen presentation pathway genes in HBV+ HCC, HBV– HCC, and non-HCC liver tissues (TCGA)

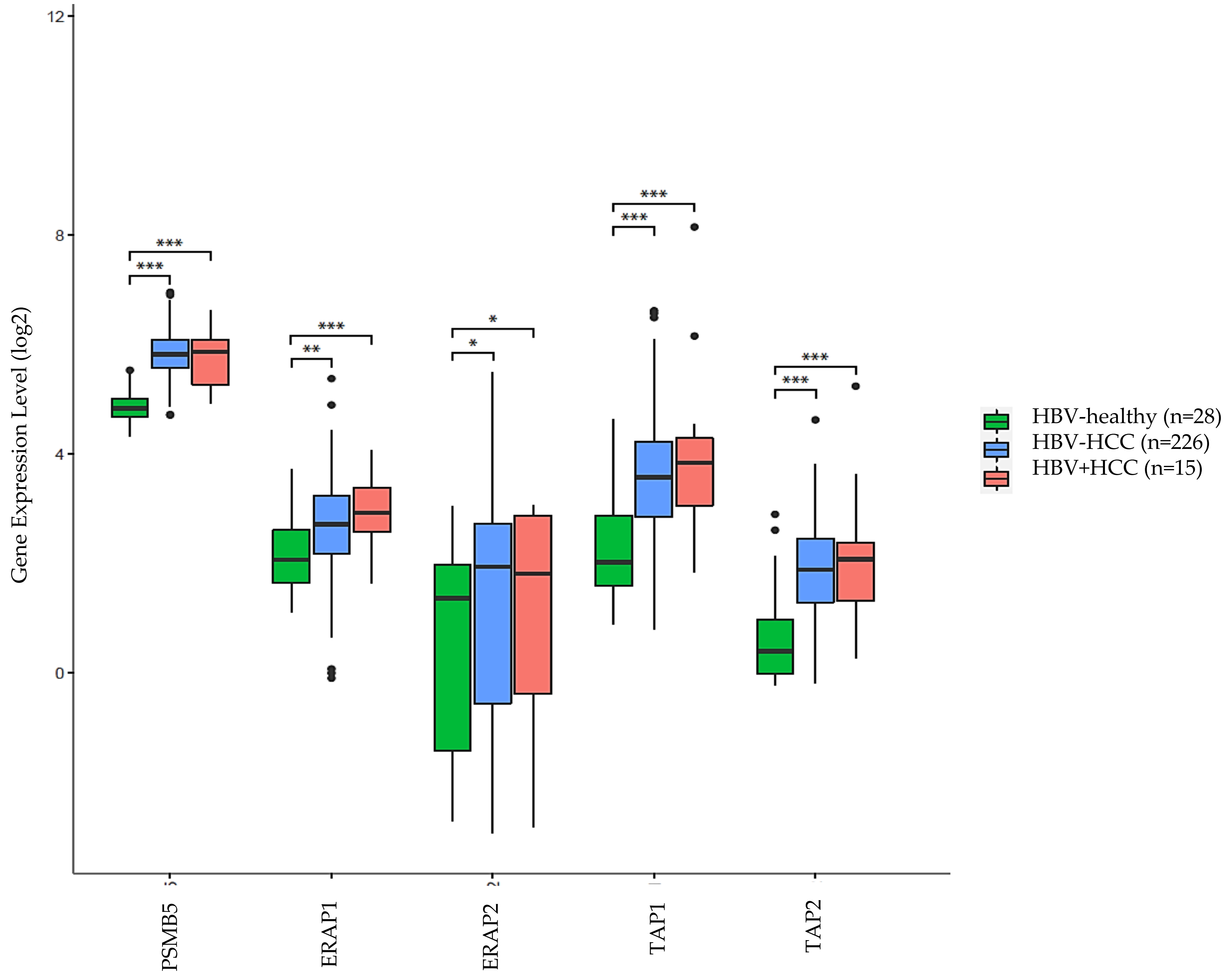

Supplement: Supplementary file 1 [file viruses-17-01454-s001.zip › Supplementary Figure S1.pdf]
