# Supplementary material for: HBV Infection Drives PSMB5-Dependent Proteasomal Activation in Humanized Mice and HBV-Associated HCC
Source: Viruses. 2025 Oct 31;17(11):1454. doi: 10.3390/v17111454 (PMC12656990; doi:10.3390/v17111454)
Supplement: Supplementary file 1 [file viruses-17-01454-s001.zip › Supplementary Table S1.pdf]

Category	Reagent / Material	Supplier (Company, City, Country)	Catalog Number	Application / Section
**ELISA Kits**	PSMB5 ELISA Kit	Elabscience, Houston, TX, USA	E-EL-H1902	Proteasome β5 quantification (2.4)
	Ubiquitin ELISA Kit	Bioassay Technology Laboratory, Shanghai, China	E4235Hu	Protein turnover (2.4)
	Protein Carbonyl ELISA Kit	Bioassay Technology Laboratory, Shanghai, China	E4746Hu	Oxidative stress (2.4)
	Proteasome β1/β2 ELISA Kits	Bioassay Technology Laboratory, Shanghai, China	E4233Hu / E4234Hu	Proteasome subunit analysis (2.4)
**Antibodies (Western blot)**	Rabbit anti-human PSMB5	Enzo Life Sciences, Farmingdale, NY, USA	BML-PW8895-0025	WB (2.9)
	Rabbit anti-Ubiquitin	Sigma Aldrich, St. Louis, MO, USA	07-375	WB (2.9)
	Mouse anti-β-actin	Santa Cruz Biotechnology, Dallas, TX, USA	sc-47778	WB (2.9)
**Antibodies (Flow cytometry)**	W6/32 hybridoma supernatant (anti-MHC I)	ATCC, Manassas, VA, USA	TIB-126	FACS (2.10)
	Donkey anti-mouse Alexa Fluor 647	Life Technologies, Carlsbad, CA, USA	A-31571	FACS (2.10)
	Zombie Violet Fixable Viability Kit	BioLegend, San Diego, CA, USA	423114	FACS (2.10)
**Molecular Biology Reagents**	RNeasy Mini Kit	Qiagen, Hilden, Germany	74104	RNA extraction (2.8)
	RNA to cDNA EcoDry Premix	Takara Bio, Kusatsu, Japan	639549	cDNA synthesis (2.8)
	LentiCRISPRv2 plasmid (blasticidin)	Addgene, Watertown, MA, USA	83480	CRISPR-Cas9 KO (2.7)
**Chemicals & Buffers**	Suc-LLVY-AMC substrate	Sigma Aldrich, St. Louis, MO, USA	S6510	Proteasome activity assay (2.3)
	DAB substrate	Dako, Copenhagen, Denmark	K3468	IHC (2.6)
